# Voltage-gated proton channels explain coccolithophore sensitivity to ocean acidification

**DOI:** 10.1073/pnas.2206426119

**Published:** 2022-06-10

**Authors:** Peter von Dassow

**Affiliations:** ^a^Departamento de Ecología, Facultad de Ciencias Biológicas, Pontificia Universidad Católica de Chile, 8331150 Santiago, Chile;; ^b^Instituto Milenio de Oceanografía, 4070386 Concepción, Chile;; ^c^Dipartimento Ecologia Marina Integrata, Stazione Zoologica Anton Dohrn, 80121 Naples, Italy

Coccolithophores are unicellular photosynthetic plankton that perform extraordinary feats in ionic homeostasis to fabricate intricate nano-patterned plates made of calcium carbonate (CaCO_3_) crystals called coccoliths (1). Outside marine science communities, coccolithophores are less known than animal calcifiers such as shellfish or the cnidarians that form coral reefs. However, coccolithophores are one of Earth’s greatest biological producers of CaCO_3_. The production and sinking of coccoliths play complex roles in ocean carbon cycles, helping carry organic carbon to the deep sea as well serving on a geological scale to help the ocean buffer CO_2_ fluctuations (2, 3). Unlike other calcifying organisms, where precipitation of CaCO_3_ is extracellular, coccolithophores calcify in special intracellular Golgi-derived coccolith vesicles. To do this, they maintain among the greatest fluxes of Ca^2+^ and H^+^ known for any cell ions which would be toxic if allowed to accumulate in the cytoplasm (1). In PNAS, Kottmeier et al. (4) demonstrate how they rely on voltage-gated proton channels to expel H^+^ released by CaCO_3_ precipitation, which also offers a way forward to resolving disparate results from two decades of research on coccolithophore sensitivity to ocean acidification.

Approximately a third of human CO_2_ emissions are absorbed by the ocean, resulting in ocean acidification. As CO_2_ dissolves in the sea it reacts with water to form carbonic acid, generating H^+^ (decreasing pH) and perturbing a set of interlocked equilibria involving CO_2_, HCO_3_^−^, CO_3_^2−^, H^+^, and Ca^2+^ by increasing [HCO_3_^−^], decreasing [CO_3_^2−^], and lowering the saturation states of alternative forms of CaCO_3_ (5). The inorganic chemistry is complex but comparatively well known. The response of calcifying organisms should be simple to predict if it depended only on the tendency of CaCO_3_ to precipitate or dissolve in seawater: Organisms such as coccolithophores which produce calcite, the more stable form of CaCO_3_, should be less sensitive to ocean acidification compared to organisms like many corals which produce less-stable forms such as aragonite.

Instead, the sophisticated and mysterious cell physiology of coccolithophores—with complex and energetically costly fluxes of Ca^2+^, H^+^, HCO_3_^−^, and CO_2_, as well as the possibility that excess CO_2_ and H^+^ can be consumed by photosynthesis in the chloroplasts—has made their sensitivity to ocean acidification far from obvious. Two decades of research (6), involving experiments in the laboratory and field, as well as looking at the coccolith fossil record through past changes in ocean pH and CO_2_ and observing how modern coccoliths respond to environmental gradients in carbonate chemistry, often gave confusing results. Although publications tended to find that ocean acidification had a negative effect (7), many contradictory findings were reported (8, 9). In the laboratory, contrasting results have been observed not only among different species but even among different genotypes of the same species, where some strains are resistant and others highly sensitive to ocean acidification (10).

The study of Kottmeier et al. (4) offers a mechanistic breakthrough, showing how one component of coccolithophore ion flux control, voltage-gated proton (H_v_) channels, can determine coccolithophore sensitivity to ocean acidification. Students of biology learn about voltage-gated ion channels specific for Na^+^, K^+^, or Ca^2+^ for their roles in the action potentials of animal nerve and muscle cells and subsequent outputs (neurotransmitter release or contraction). The existence of voltage-gated channels specific to H^+^ first came to light in another unicellular planktonic organism, dinoflagellates, where a H^+^ action potential mediates a bioluminescent flash (11, 12), and were later found in animals (13). Electrophysiology guided by genomics and transcriptomics indicated that coccolithophores contain H_v_ homologs (14). This earlier study, in the same laboratory as the present Kottmeier et al. study, suggested that coccolithophore H_v_ channels might help pH regulation during calcification, but it still was not clear how that worked or affected sensitivity to ocean acidification.

Most coccolithophore studies focus on the cosmopolitan and abundant species *Emiliania huxleyi*, whose vast blooms turn the sea white in the North Atlantic and large bands just north of the Southern Ocean (3). Kottmeier et al. (4) instead studied the heavily calcified species *Coccolithus braarudii*, which fixes more C in the form of CaCO_3_ than as particulate organic carbon (e.g., cell components), so it contributes disproportionately to CaCO_3_ fluxes compared to more lightly calcified species and exhibits the highest demands for calcification-related ion fluxes.

Kottmeier et al. (4) show that exposure of *C. braarudii* to ocean acidification conditions predicted for the next century caused specific defects in coccolith fabrication due to loss of H_v_ activity. H^+^ efflux, rather than the K^+^ efflux as in other eukaryotes, appears to determine the voltage (potential) across the plasma membrane in *C. braarudii* (1, 14), and the activation voltage of the H_v_ channels are near this membrane potential. This means that intracellular pH is strongly affected by fluctuations in extracellular pH (4). This normally is an effective system for pH homeostasis in open oceans, where seawater pH is very stable, averaging near 8.2 in preindustrial times (5). The difference between pH_cyt_ and seawater pH results in a proton motive force (pmf) pushing H^+^ out of the cell, and H_v_ channels permit very rapid disposal of H^+^ generated during calcification to a vast extracellular seawater sink (Fig. 1, *Left Side*).

Ocean acidification lowers the difference between pH_cyt_ and seawater pH, altering the pmf so H_v_ channels can no longer provide pH control. Under these conditions, *C. braarudii* actually maintained tighter control of cytoplasmic pH in response to extracellular pH fluctuations, because H_v_ channels remained closed while active H^+^ transport continued, probably involving Na^+^/H^+^ exchangers pushing H^+^ out of the cell (Fig. 1) and V-ATPases sequestering H^+^ in intracellular compartments, both energy-consuming mechanisms (4). Pharmacological H_v_ blockers replicated the ocean acidification-specific coccolith defect. These results suggest that continued CaCO_3_ precipitation in the coccolith vesicle depends on dynamic control of H^+^ flux offered by H_v_ channels, a model which explains how increased [H^+^] of ocean acidification directly causes negative impacts on calcification in these coccolithophores even when seawater remains supersaturated with respect to calcite.

The authors hypothesize that the dependence of a coccolithophore on H_v_ channels for pH homeostasis varies with how heavy the coccoliths produced are (the rate of calcification determining how rapidly H^+^ must be effluxed). Some contradictions remain to be addressed. For example, wide variation exists in the degree and rate of calcification within the single species *E. huxleyi*, and the strains reported to be most sensitive to ocean acidification were also the least-calcifying strains that produced the most delicate coccoliths (10). Lower dependence on H^+^ efflux through H_v_ channels could involve mechanisms such as higher photosynthetic consumption of H^+^ or relying more on active H^+^ pumping out of the cytosol. The authors’ discussion of alternative mechanisms that coccolithophores might use to control H^+^ fluxes, which may become adaptive as ocean pH drops, might also offer a path to explaining the variety of responses to ocean acidification seen in different species and strains.

A wide range of selective functions for coccoliths have been proposed, including protection against grazing, a CO_2_-concentrating mechanism, and modulation of light, but current evidence is inconclusive (15), meaning some ecological consequences remain unresolved. While the present study did not address this issue, it will help in refining estimates of the metabolic costs of calcification that must be offset by whatever benefits are provided. Also, lighter coccoliths should decrease CaCO_3_ flux to the seafloor and also might decrease organic C flux and midwater O_2_ levels if more organic material, without the ballast of heavy coccoliths, is respired at shallower depths before reaching the seafloor (16).

Community genomic and transcriptomic sequencing efforts of the 2000s led by the Joint Genome Initiative in the United States (17) and Genoscope in France (18) allowed identifying the H_v_ homologs. However, the present study faced a technical limitation that genetic transformation is still not possible in *C. braarudii*, and success in related species (19) has not yet led to widely successful protocols. Conclusions relied on experiments with pharmacological inhibitors of H_v_ channels such as Zn^2+^ and 2-guanidinobenzimidazole (2-GBI). Although the authors did not detect other effects on cell physiology from either inhibitor, there are always limits to the specificity of pharmacological agents. Also, evaluating whether the two distinct coccolithophores’ H_v_s have different roles must await reliable methods for genetic manipulation in these organisms (e.g., CRISPR or RNA interference). This highlights how the recent push for genetic tool development for marine protists (20) could provide essential insight to challenging questions in global biogeochemistry under climate change.

**Fig. 1.**
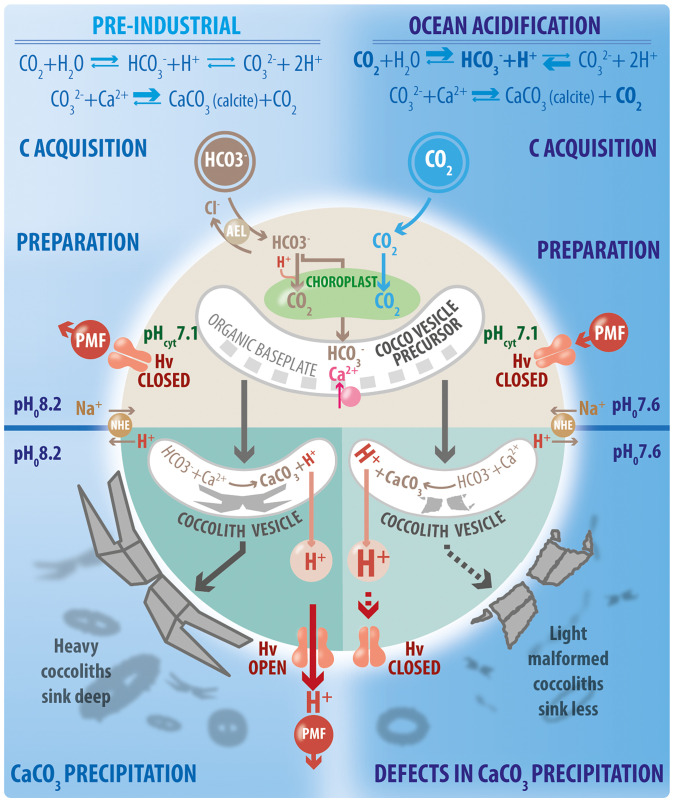
The effects of ocean acidification on coccolithophore calcification is more complex than seawater chemistry predicts, since they precipitate CaCO_3_ inside the cell. Higher CO_2_ absorbed by the sea increases H^+^ and pushes equilibria of inorganic carbon species toward HCO_3_^−^. As large regions of the surface ocean become undersaturated with respect to aragonite, a less-stable form of CaCO_3_, most will remain supersaturated with respect to calcite, the most stable form precipitated by coccolithophores. The Kottmeier et al. (4) study explains why they are still sensitive to ocean acidification. H_v_ channels are closed as the coccolith vesicle precursor accumulates Ca^2+^, HCO_3_^−^, and organic baseplate for crystal growth. Calcite precipitation in the mature vesicle creates excess H^+^. Photosynthesis uses dissolved CO_2_ directly or converts HCO_3_^−^ to CO_2_ in the chloroplast, consuming H^+^, but, under normal conditions, H_v_ channels open, allowing H^+^ efflux to follow the pmf. Heavy coccoliths are formed that sink deep into the ocean. Under ocean acidification, the pmf is reversed. Even if an outward pmf were restored with further drop in pH_cyt_, H_v_ channels remain inactive. Higher CO_2_ under ocean acidification may decrease use of HCO_3_^−^ by the chloroplast, exacerbating H^+^ loads due to calcification. Only energy-consuming H^+^ transport such as Na^+^/H^+^ exchangers (NHE) or V-ATPases (not shown) can maintain cytosol pH. Slow H^+^ efflux negatively affects crystal growth, causing lighter, malformed coccoliths which sink less, decreasing CaCO_3_ export to the deep sea. The pH values are illustrative, consistent with the model of Kottmeier et al. (4), and within measured ranges. AEL, anion exchanger-like transporter of HCO_3_^−^; Hv, voltage-gated H^+^ channel.

## References

[1] A. R. Taylor, C. Brownlee, G. Wheeler, Coccolithophore cell biology: Chalking up progress. Annu. Rev. Mar. Sci. 9, 283–310 (2017). 10.1146/annurev-marine-122414-034032 27814031

[2] A. Ridgwell, R. Zeebe, The role of the global carbonate cycle in the regulation and evolution of the Earth system. Earth Planet. Sci. Lett. 234, 299–315 (2005).

[3] W. M. Balch, The ecology, biogeochemistry, and optical properties of coccolithophores. Annu. Rev. Mar. Sci. 10, 71–98 (2018). 10.1146/annurev-marine-121916-063319 29298138

[4] D. M. Kottmeier, , Reduced H^+^ channel activity disrupts pH homeostasis and calcification in coccolithophores at low ocean pH. Proc. Natl. Acad. Sci. U.S.A. 119, e2118009119 (2022). 35522711 10.1073/pnas.2118009119 PMC9171652

[5] R. A. Feely, S. C. Doney, S. R. Cooley, Ocean acidification: Present conditions and future changes in a high-CO_2_ world. Oceanography 22, 36–47 (2009).

[6] U. Riebesell , Reduced calcification of marine plankton in response to increased atmospheric CO2. Nature 407, 364–367 (2000). 11014189 10.1038/35030078

[7] J. Meyer, U. Riebesell, Reviews and syntheses: Responses of coccolithophores to ocean acidification: A meta-analysis. Biogeosciences 12, 1671–1682 (2015).

[8] M. D. Iglesias-Rodriguez , Phytoplankton calcification in a high-CO_2_ world. Science 320, 336–340 (2008). 18420926 10.1126/science.1154122

[9] L. Beaufort , Sensitivity of coccolithophores to carbonate chemistry and ocean acidification. Nature 476, 80–83 (2011). 21814280 10.1038/nature10295

[10] M. Müller, T. Trull, G. Hallegraeff, Differing responses of three Southern Ocean *Emiliania huxleyi* ecotypes to changing seawater carbonate chemistry. Mar. Ecol. Prog. Ser. 531, 81–90 (2015).

[11] T. Nawata, T. Sibaoka, Coupling between action potential and bioluminescence in Noctiluca: Effects of inorganic ions and pH in vacuolar sap. J. Comp. Physiol. 134, 137–149 (1979).

[12] S. M. E. Smith , Voltage-gated proton channel in a dinoflagellate. Proc. Natl. Acad. Sci. U.S.A. 108, 18162–18167 (2011). 22006335 10.1073/pnas.1115405108 PMC3207696

[13] T. E. DeCoursey, V. V. Cherny, Potential, pH, and arachidonate gate hydrogen ion currents in human neutrophils. Biophys. J. 65, 1590–1598 (1993). 7506066 10.1016/S0006-3495(93)81198-6 PMC1225885

[14] A. R. Taylor, A. Chrachri, G. Wheeler, H. Goddard, C. Brownlee, A voltage-gated H+ channel underlying pH homeostasis in calcifying coccolithophores. PLoS Biol. 9, e1001085 (2011). 21713028 10.1371/journal.pbio.1001085 PMC3119654

[15] F. M. Monteiro , Why marine phytoplankton calcify. Sci. Adv. 2, e1501822 (2016). 27453937 10.1126/sciadv.1501822 PMC4956192

[16] M. Hofmann, H. J. Schellnhuber, Oceanic acidification affects marine carbon pump and triggers extended marine oxygen holes. Proc. Natl. Acad. Sci. U.S.A. 106, 3017–3022 (2009). 19218455 10.1073/pnas.0813384106 PMC2642667

[17] B. A. Read ; *Emiliania huxleyi* Annotation Consortium, Pan genome of the phytoplankton *Emiliania* underpins its global distribution. Nature 499, 209–213 (2013). 23760476 10.1038/nature12221

[18] P. von Dassow , Transcriptome analysis of functional differentiation between haploid and diploid cells of *Emiliania huxleyi*, a globally significant photosynthetic calcifying cell. Genome Biol. 10, R114 (2009). 19832986 10.1186/gb-2009-10-10-r114 PMC2784329

[19] H. Endo , Stable nuclear transformation system for the coccolithophorid alga *Pleurochrysis carterae*. Sci. Rep. 6, 22252 (2016). 26947136 10.1038/srep22252 PMC4779993

[20] D. Faktorová , Genetic tool development in marine protists: Emerging model organisms for experimental cell biology. Nat. Methods 17, 481–494 (2020). 32251396 10.1038/s41592-020-0796-x PMC7200600

